# Clinical activity is an independent risk factor of ischemic heart and cerebrovascular arterial disease in patients with inflammatory bowel disease

**DOI:** 10.1371/journal.pone.0201991

**Published:** 2018-08-31

**Authors:** Guillaume Le Gall, Julien Kirchgesner, Mohamed Bejaoui, Cécilia Landman, Isabelle Nion-Larmurier, Anne Bourrier, Harry Sokol, Philippe Seksik, Laurent Beaugerie

**Affiliations:** 1 Département de gastroentérologie, Hôpital Saint Antoine, AP-HP, Paris, France; 2 UMR-S 1136, INSERM & UPMC Univ Paris 06, Paris, France; 3 INSERM, ERL 1057 INSERM Unité Mixte de Recherche 7203 and Groupe de Recherche Clinique–03, Sorbonne Université, Paris, France; Kurume University School of Medicine, JAPAN

## Abstract

**Background and aims:**

In inflammatory bowel disease (IBD), the impact of established cardiovascular risk factors and disease-related factors on the risk of acute arterial events is still unclear. We aimed to identify risk factors of acute arterial events in patients with IBD.

**Methods:**

All consecutive patients followed at Saint-Antoine Hospital between 1996 and 2015 with subsequent occurrence of acute arterial events (acute coronary syndrome or ischemic stroke) were identified. Traditional cardiovascular risk factors, treatment exposure, systemic inflammation (mean serum CRP level greater than or equal to 5 mg/L) and IBD clinical activity were assessed. A nested case-control study was performed including cases and controls without arterial ischemic event, matched on age, gender, and disease extent.

**Results:**

A total of 30 patients (median age at acute vascular event occurrence: 42 years (interquartile range: 25–59)) developed acute coronary syndrome (n = 22) or ischemic stroke (n = 8). In univariate analysis, clinical disease activity and the persistence of systemic inflammation, diabetes, dyslipidemia and hypertension were significantly associated with an increased risk of acute arterial events. Neither protective nor aggravating effects associated with treatment exposure were identified. In multivariate analysis, the presence of diabetes (Odds ratio (OR): 14.5, 95% confidence interval (CI): 1.1–184.7) and clinical disease activity (OR: 10.4, 95% CI: 2.1–49.9) remained significantly associated with the risk of acute arterial event.

**Conclusion:**

Disease activity may have an independent impact on the risk of acute arterial events in patients with IBD. These findings may highlight new potential benefits of optimizing anti-inflammatory treatment in patients with persisting clinical activity.

## Introduction

Inflammatory bowel disease (IBD) including Crohn’s disease and ulcerative colitis are characterized by chronic intestinal and systemic inflammation, while chronic systemic inflammation plays a central role in atherogenesis and consequently increases the risk of atherosclerosis [1,2]. In patients with IBD, it has been constantly reported an increased risk of acute arterial events, including ischemic heart disease, cerebrovascular disease and peripheral artery disease [3–6]. In addition, this increased risk was also reported in patients with other systemic inflammatory diseases, such as rheumatoid arthritis [7,8]. However, the association between chronic systemic inflammation and the risk of acute arterial events remains unclear in patients with IBD. Two studies based on medico-administrative databases concluded that IBD disease activity was an independent predictor of acute arterial events [5,6]. However, the disease activity was assessed trough indirect markers, including hospitalizations, surgical procedures, and treatment exposure [6,9]. Systemic inflammation was not monitored in these two studies. Other studies on persons without IBD concluded that systemic inflammation based on serum C-reactive protein (CRP) level predicts ischemic heart disease, cerebrovascular disease, and cardiovascular death, even after adjustment for the traditional cardiovascular risk factors [10,11]. Hence, the impact of intestinal and systemic inflammation on the risk of acute arterial events in patients with IBD remains unclear and needs further investigation.

The aim of our study was to assess the impact of intestinal and systemic inflammation on the risk of acute arterial event in patients with IBD.

## Methods

### Data source

The MICISTA database is a cohort of patients with IBD followed by the same medical team of gastroenterologists at the Rothschild Hospital from 1994 to 2002 and at the Saint Antoine Hospital since 2003 in Paris [11]. The prospective data collection began in 1994 and has since continued. The diagnosis of IBD was confirmed by endoscopy, pathology and imaging. Recorded variables include: (1) demographic characteristics; (2) disease phenotype, initial and cumulative disease extension; (3) clinical disease activity; (4) treatment related o IBD; (5) associated diseases, notably cancer and cardiovascular disease. The study was approved by the French Data Protection Supervisory Authority (Commission Nationale de l’Informatique et des Libertés [CNIL—1 104 603]).

### Patients with acute arterial event

All patients, aged 18 or older with an occurrence of acute arterial event between 1996 and 2015 were identified from the MICISTA database. Only patients with regular follow-up were analyzed. Regular follow-up was defined as a follow-up greater than one year and at least one visit per year in our IBD unit.

Patients with an occurrence of acute arterial event prior to the diagnosis of IBD were excluded. Cases were selected based on a variable corresponding to the vascular manifestation. Among these patients, the type of arterial ischemic event was assessed through medical records. Acute coronary syndrome was defined by chest pain with electrocardiogram modification, documented elevation in cardiac troponin and evidence of a culprit lesion on coronary [12]. Stroke was defined by neurologic clinical modification and confirmed ischemia in magnetic resonance imaging [13]. Patients were excluded in case of active neoplasia or occurrence of neoplasia in the previous 5 years before acute arterial event, and in the absence of clinical or biological monitoring prior to the date of acute arterial event occurrence.

### Nested case-control study

Since the level of systemic inflammation is not reported in the MICISTA database, a nested case-control study assessing the impact of systemic inflammation was conducted in the MICISTA cohort. As defined previously, patients with an occurrence of acute arterial event were selected as cases. Patients without an occurrence of acute arterial event were selected as controls. Matching criteria were sex, the date of birth (within five years), the date of diagnosis (within five years), and the phenotype of IBD (Crohn's disease or ulcerative colitis), patients with unclassified IBD were included in the ulcerative colitis group. All variables were assessed from MICISTA database and medical records of cases and matched controls in the year prior to the occurrence of acute arterial event.

Variable related to systemic and intestinal inflammation were assessed as follows: clinical activity was characterized according to the MICISTA database classification [14–16], (0: quiescent disease; 1: minor symptoms (chronic or intermittent gastrointestinal symptoms) with questionable link with IBD activity, and without impact on professional life; 2: mild symptoms (chronic or intermittent gastrointestinal symptoms) whose link with IBD is likely, but without impact on professional life; 3: flare or chronic disease activity affecting professional life; 4: hospitalization related to IBD disease activity; 5: surgical procedure related to IBD disease activity) Clinical activity was defined as the most severe condition within the year preceding the acute arterial event. Two groups were formed: the quiescent disease group (0) and the clinical active disease group (1 to 5). Average CRP> 5mg / L, platelet count, and hemoglobin level were recorded in the year and in the three years preceding the arterial ischemic event. Traditional cardiovascular risk factors (arterial hypertension, dyslipidemia, diabetes, active smoking, obesity and age) were also collected. Diagnoses of diabetes, dyslipidemia, and arterial hypertension were based on the medical file information on exposure to anti-diabetics, lipid-lowering drugs and anti-hypertensive drugs, respectively. Patients were considered exposed to IBD-related treatment (corticosteroids, 5 amino-salicylates, thiopurines, methotrexate, and anti–tumor necrosis factor agents [anti-TNFs]) in case of any treatment exposure within the year preceding the acute arterial event. We chose arbitrarily as the marker of the impact of exposure to corticosteroids the highest dose (mg per day) used within the year preceding the acute arterial event in cases.

The collection of variables of interest was carried out from the MICISTA database (phenotype of the disease, clinical activity, smoking status, type of treatment, age at diagnosis) and from medical records (date and type of acute arterial event, traditional cardiovascular risk factors). In matched controls, all variables were collected after the same time interval as in cases between the date of diagnosis and the date of the acute arterial event occurrence.

### Statistical analysis

Quantitative variables were compared by a Wilcoxon-Mann-Whitney test. Qualitative variables were compared by a Chi-2 or Fisher test, as appropriate. The statistically significant difference level was defined as p <0.05 (bilateral test). An univariate conditional logistic regression was performed in order to quantify the relationship between occurrence of acute arterial event and the variables of interest. All variables significantly associated with occurrence of an acute arterial event with p-value <0.20 in univariate conditional logistic regression and all traditional cardiovascular risk factors (due to their established association with acute arterial event) were included in a multivariate logistic regression model with a backward variable elimination procedure to assess the strength of the associations while controlling for possible confounding variables.

## Results

### Characteristics of patients

**Fig 1:**
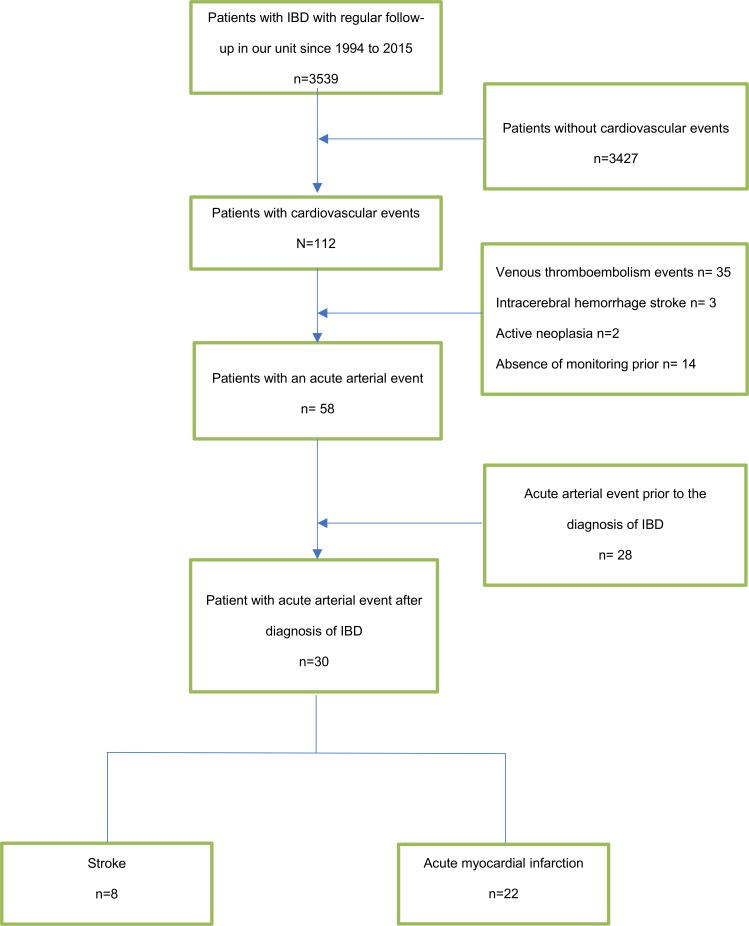
Flow chart.

Among 8164 patients identified with IBD in the 1994–2015 MICISTA database, 3539 patients were regularly followed in our unit during the study period. Among them, an acute vascular event occurred in 112 patients. Additionally, 82 of these patients were not eligible for inclusion: 35 patients had a venous thromboembolic event, 3 patients had a hemorrhagic stroke, 2 patients had active neoplasia, 14 patients did not have monitoring prior the acute arterial event and 28 patients had acute arterial event prior to the diagnosis of IBD. In total, 8 patients with an ischemic stroke and 22 patients with acute coronary syndrome after the diagnosis of IBD were finally included in the study (Fig 1).

As expected, no statistically significant differences were observed between the two groups for any of the matching criteria. Overall, patients were predominantly male (53 [59%]) with a median age at IBD diagnosis of 47 years (interquartile range, IQR: 32–57) and 43 years (IQR: 32–54) in cases and controls (p = 0.27), respectively. The disease location was predominantly colonic or ileocolonic for Crohn's disease and distal or left-sided for ulcerative colitis according to the Montreal classification. Patient characteristics according the occurrence of acute arterial event are provided in Table 1.

**Table 1:** Characteristics of patients included in the case-control study.

			Case n = 30	Controls n = 60	p-value
General characteristics					
	Age at diagnosis of IBD (years)		41.9(25.3–58.7)	43.3(31.7–54.6)	
	Male		18 (60)	35 (58.3)	0.27
Type of IBD					
	Crohn’s disease		20 (66.7)	42 (70.0)	
		ileum	5 (25.0)	11 (26.2)	
		colonic	8 (40.0)	19 (45.2)	
		ileocolonic	7 (35.0)	12 (28.6)	
	Ulcerative colitis		8 (26.7)	17 (28.3)	
		Proctitis	3 (37.5)	6 (35.3)	
		Left-sided UC	4 (50.0)	9 (52.9)	
		Extensive UC	1 (12.5)	2 (11.8)	
		Unclassified IBD	2 (6.7)	1 (5.9)	
Type of ischemic event					
	Stroke		8 (26.7)		
	Acute coronary syndrome		22 (73.3)		
					

Results are expressed as median (interquartile range) or n (%).

Among patients with of acute arterial event, the median interval between IBD diagnosis and occurrence of acute arterial event was 15.4 years (IQR: 1.9–24.3). Regarding traditional cardiovascular risk factors, 23.3% (N = 7) of patients had diabetes, 33.3% (N = 10) had dyslipidemia, 63.3% (N = 19) were older than 50 years at the occurrence of acute arterial event. In the year prior to acute arterial event, 46.7% (N = 14) reported active smoking, 33.3% (N = 10) had active chronic disease, 23.3% (N = 7) had been hospitalized related to IBD disease activity in the year prior to acute arterial event. Regarding treatment exposure, 6.7% (N = 2), 23.3% (N = 7), 30.0% (N = 9) of patients were exposed to anti-TNFs, immunosuppressants (thiopurines and methotrexate), and corticosteroids, respectively.

### Impact of systemic inflammation

In univariate analysis, clinically active IBD was associated with an increased risk of acute ischemic event (Odds ratio (OR): 12.3, 95%CI: 2.8–53.6). Regarding systemic inflammation, the presence of an average CRP level greater than 5mg/L in the year or in the preceding 3 years were all associated with an increased risk of acute ischemic event (OR: 3.2, 95%CI: 1.2–8.5). No statistically significant differences between cases and controls were observed for exposure to immunosuppressants, anti-TNFs or corticosteroids. As expected, diabetes, dyslipidemia and arterial hypertension were associated with an increased risk of acute arterial event. For univariate regression analysis refer to Table 2.

**Table 2:** Univariate regression analysis.

* *	* *	*Cases*	*Controls*	*Odds ratio*	*P*
* *	* *	*n = 30*	*n = 60*	*(CI 95%)*	* *
Clinical activity		25 (83.3)	26 (43.3)	10.0 (2.2–44.3)	0,002*
Average CRP level over 3 years >5mg/L		25 (83.3)	41 (68.3)	2.5 (1.1–6.4)	0,04*
Average CRP level the year >5mg/L		18 (60)	21 (35)	3.2 (1.2–8.5)	0,02
Platelet count** (×10^9^/L)		259 (209–312)	250 (209–308)	1.0 (0.9–1.1)	0.83
Smoking status		14 (46.7)	24 (40)	1.3 (0.3–5.5)	0,67*
Diabetes		7 (23.3)	2 (3.3)	11.2 (1.4–92.4)	0,02*
Dyslipidemia		10 (33.3)	8 (13.3)	5.8 (1.2–28.5)	0,03*
Arterial hypertension		16 (53.3)	15 (25)	3.6 (1.2–10.3)	0,02*
Immunosuppressants (methotrexate, azathioprine)					
Corticosteroids					
	0	21 (70)	44 (73.3)	Ref	-
	0-20mg/j	8 (26.7)	15 (25)	1.1 (0.4–3.0)	0.98
	> 20mg/j	1 (3.3)	1 (1.7)	1.1 (0.1–17.6)	

Results are expressed as median (interquartile range) or n (%). CI 95%: 95% Confidence interval

*: variables included in multivariate analysis

** Odds ratio for 10 000 units increase

In multivariate analysis, the presence of diabetes (OR: 14.5, 95% CI: 1.1–184.7) and clinical disease activity (OR: 10.4, 95% CI: 2.1–49.9) remained significantly associated with the risk of acute arterial event (Table 3).

**Table 3:** Multivariate regression analysis.

	Odds Ratio (95% CI)	p


Diabetes	14.5 (1.1–184.7)	0.04
Clinical activity	10.4 (2.1–49.9)	0.01

Variables included in the model: diabetes, dyslipidemia, hypertension, smoking status, 3-year CRP mean, clinical activity

## Discussion

In the present nested case-control study, clinical disease activity was associated with an increased risk of acute arterial events in patients with IBD. Chronic systemic inflammation was associated with an increased risk of acute arterial events in univariate analysis but not in multivariate analysis. Traditional cardiovascular risk factors were expectedly associated with an increased risk of acute arterial event.

We reported a trend between the risk of acute arterial event and chronic systemic inflammation defined by mean CRP level greater than 5mg/L in the year or in the 3 years preceding acute arterial event. This association was statistically significant in univariate regression analysis although not significant in multivariate analysis. This finding is in accordance with previous studies reporting elevated levels of CRP as an independent cardiovascular risk factor [9,17]. It also suggest that the persistence of residual acute or chronic inflammation is associated with an increased risk of acute arterial events and supports the concept of zero tolerance of residual inflammation, in order to avoid long-term ischemic vascular complications.

The main strength of our study is that the entire patient population was drawn from the MICISTA cohort, ensuring accurate assessment of risk factors and IBD clinical activity based on our access to over 20 years follow-up and full medical records for all included patients. Additionally, studies assessing the risk of acute arterial events in patients with IBD did not accurately assess systemic inflammation[18]. In a 2015 US study, the introduction of corticosteroids in patients with IBD reduced the risk of arterial ischemic event (19). The group of patients who experienced an acute ischemic event, in this study, had more cardiovascular risk factors than the patients in our study. However, as key clinical and biological data did not allow the identification of risk factors, less than 20% of the patients had biological marker as CRP level[19]. This is the first study that gathers clinical activity data, systemic inflammation and IBD treatment.

No protective factors against occurrence of acute arterial events, including corticosteroid therapy, immunosuppressants or anti-TNFs were observed in our study. While these parameters may be involved in decreasing the occurrence of such events, their true associations may have been impossible to demonstrate in our study due to lack of sufficient statistical power as the sample size was small. A study published in 2014, evaluating immunomodulatory molecules used in IBD treatments ‘effect on the rigidity of arterial vascular wall, reported that systemic inflammation was significantly involved in increasing arterial rigidity. This rigidity was reduced by immunomodulatory treatments [20].

Traditional cardiovascular risk factors were associated with an increased risk of acute arterial events. Since patients with IBD are at increased risk of acute arterial events, it would seem reasonable to routinely screen for major cardiovascular risk factors in this population and to ensure their appropriate management once detected. Guidelines regarding the cardiovascular risk management in patients with rheumatoid arthritis and other forms of inflammatory joint disorders are published since 2009 [21]. However, the screening of cardiovascular risk factors is generally not part of the management algorithm for patients with chronic inflammatory bowel disease. A study published in 2011 found that among patients with IBD, less than 20% were assessed for cardiovascular risk factors, while 100% were assessed for inflammatory biomarkers. [22]. Yet, it seems necessary to assess major cardiovascular risk factors as part of management of these patients as it is performed for extra-intestinal manifestations of IBD [22]. The prevention of cardiovascular events requires management of both the cardiovascular risk factors and the background chronic inflammatory pathology.

Some limitations should be noted. The definition of disease activity was prospectively assessed according to the MICISTA database classification. Although this definition has been used in several studies [14–16], the assessment of disease activity based on scores used in clinical trials would have increased the generalizability of our findings. Second, although the definition of diabetes, dyslipidemia, and hypertension were based on related treatment exposure (antidiabetic, lipid-lowering drugs and anti-hypertensive drugs, respectively) in individual medical records, details on exposure to these drugs were not specifically collected. Similarly, coagulation factors were not collected. Since atherosclerosis is a chronic inflammatory condition of the vessel wall, inflammatory biomarkers have been shown to predict risk, monitor treatments, and guide therapy. Although several biomarkers were identified during the last years, CRP measured by a highly sensitive assay remains the most widely studied. Several studies concluded that CRP level predicts ischemic heart disease, cerebrovascular disease, and cardiovascular death even after adjustment for the traditional Framingham covariates [9,10].

In conclusion, our study demonstrates that clinically active IBD was significantly associated with an increased risk of acute arterial event in patient with IBD. This key finding of increased risk of acute arterial event among patients with clinically active disease highlights the need for effective treatment, even in patient with low-grade symptoms but with persistent biological inflammatory syndrome. Additionally, screening and management of cardiovascular risk factors appear to be essential to reducing this risk of acute arterial event. Further studies are needed to assess the beneficial effect of IBD related treatment in reducing the risk of acute arterial events.

## Supporting information

S1 File Dataset. (XLSX)

## Abbreviations

IBD: Inflammatory bowel disease
CD: Crohn's disease
UC: Ulcerative colitis
PSC: primary sclerosing cholangitis
CRC: colorectal cancer
5-ASA: 5-aminosalicylates
anti-TNFs: anti-tumor necrosis factor agents
